# Supplementary material for: Are carbon emissions trading and green financial instruments synergistic? -Comprehensive quantitative research based on content analysis
Source: PLoS One. 2024 Mar 7;19(3):e0298601. doi: 10.1371/journal.pone.0298601 (PMC10919601; doi:10.1371/journal.pone.0298601)
Supplement: S1 Data — (ZIP) [file pone.0298601.s001.zip › Data/地级市Z_F工作报告.zip!/╣π╬≈/╬α╓▌╩╨/└┤╫╘╣1⁄2╓┌║┼í╛┬φ┐╦╩2╛▌═°í┐_2017.docx]

代表：

现在，我代表市人民政府向大会作政府工作报告，请予审议，并请市政协各位委员提出意见。

2016年工作回顾

过去的一年，面对经济下行压力持续加大的严峻形势，我们在自治区党委、政府和市委的坚强领导下，在市人大、市政协的监督支持下，坚持以改革创新和向东开放为动力，以推进供给侧结构性改革为主线，全力打好稳增长和脱贫攻坚战，保持了经济基本面和社会大局总体稳定，实现了“十三五”良好开局。

——经济运行保持稳健。预计，全市完成地区生产总值1180亿元，增长7.7%；规模以上工业增加值610亿元，增长9.5%；固定资产投资1169亿元，增长12%；财政收入127.6亿元，增长3.1%；社会消费品零售总额396亿元，增长8.5%。总体上看，主要经济指标增速保持在合理区间，经济发展实现稳中有进、稳中趋好。

——质量效益稳步提升。

工业总产值超亿元的企业发展到230家，亿元企业实现工业总产值超过2000亿元，工业企业利润增长6%。

投资结构不断优化，工业投资突破550亿元，高新产业投资增长20%以上。万元工业增加值能耗降低3.5%。

财政结构进一步优化，非税收入占比下降2.2个百分点。

——创新创业取得实效。全市有效发明专利拥有量超过500件，每万人口发明专利拥有量增长48%以上。新增自治区企业技术中心5家，14家企业通过国家高新技术企业认定。建成梧州学院国家级众创空间和4家科技企业孵化器，全市在孵企业达136家。全市新增注册企业3700多家、个体户1.3万多户。

——民生福祉不断改善。全年民生支出达187亿元，占公共财政预算支出的81.9%。城镇居民、农村居民人均可支配收入分别增长9%和10%。实现了4.77万贫困人口脱贫、59个贫困村和龙圩区脱贫摘帽。棚户区改造开工建设1.6万多套，完成农村危房改造7110户。大病保险实现政策全覆盖，实现患者出院基本医保、大病保险和医疗救助一站式结算，实现区内异地就医购药刷卡直接结算。发展成果更多更公平地惠及广大百姓，人民群众的获得感不断提升。

一年来，我们重点抓了八个方面工作：

全力稳增长保发展态势。

围绕自治区下达的“六稳”目标任务精准施策，保持了经济平稳发展态势。稳投资方面，坚持以“三年一工程”为抓手，统筹推进西江经济带基础设施建设大会战、城市建设三年提升工程和“两区一城”项目建设，全年安排重大项目510个，累计完成投资417亿元。

稳工业方面，持续开展“千名干部进千企”活动，帮助企业降成本、减负担，为企业减少电费负担1.2亿多元，推动4家企业在新三板挂牌、60多家企业在区域性股权交易中心挂牌，通过实施“惠企贷”累计为41家企业提供贷款3亿多元。

稳农业方面，粮食总产量达82.2万吨，蔬菜播种面积达120万亩，水果种植面积达75.7万亩，全市完成农林牧渔业总产值232亿元，增长4.2%。

稳消费方面，组织开展消费购物节等系列大型促销展销活动，启动实施电子商务“十百千万”工程，培育新增限上企业40多家。稳进出口方面，引导和组织外贸企业参加大型交易博览会开拓国际市场，落实梧州至香港定期班轮、口岸集装箱补助等优惠政策，促进外贸出口数据回流。稳财政方面，加强税源培育和收入组织，大力争取上级资金补助，积极盘活存量资金，改善收支结构，重点保民生支出，完成财政八项支出166亿元，增长13.4%。

大力推动产业转型升级。深入实施现代特色农业产业提升行动。打响特色优势农业产业品牌，富硒农产品生产面积达1万多亩，岑溪古典鸡、藤县江口荔枝分别获得国家地理标志登记和农业部农产品地理标志登记；市本级成立茶产业办公室和梧州六堡茶股份有限公司，苍梧县被评为“全国十大魅力茶乡”。加快现代特色农业示范区建设步伐，建成自治区、市、县、乡四级示范区27个，其中蒙山县东乡桔园、岑溪市古典三黄鸡生态养殖基地、万秀区思良江休闲农业区获评为自治区级现代农业（核心）示范区。积极培育新型农业经营主体和新型职业农民，新增农民专业合作社455家、家庭农场107家。发展壮大循环经济。

围绕延伸再生资源、再生不锈钢制品产业链，引进产业项目20多个，开工建设了金海不锈钢精深加工园中园、年产3万吨金属精密压铸、梧州再制造基地等产业项目，两大产业实现产值近1000亿元。加大新兴产业培育力度。开工建设了中盟医药产业园，市静脉产业园成为首个签约落地PPP项目，新华锂离子汽车动力电池、20兆瓦光伏发电一期工程等项目竣工投产。

推动服务业加快发展。推进市商贸物流园区等项目建设，毅德商贸物流城开始对外营业。

加大旅游宣传推广和景区景点建设力度，成功举办了2016年广西岭南风情旅游文化周活动，苍海旅游区成功申报国家4A级景区。全市接待游客超过1700万人次，同比增长13%；实现国内旅游收入192亿元，同比增长25%。

着力打牢基础增强发展后劲。

交通方面。稳步推进铁路建设，赤水铁路专用线完工并投入使用，岑溪至罗定铁路复工相关工作扎实推进。

加快高速公路建设，岑溪至水汶高速公路建成通车，梧州至柳州高速公路、环城高速公路等项目进展顺利。

梧州机场迁建工程全面开工，桂江（莲花大桥至桂江河口）航道整治工程完工。

城市建设方面。组织实施城市建设提升7大类168个项目，全年完成投资超过90亿元。西江三桥可望今年4月建成通车，启动了防洪工程建设会战，全年完成城市基础设施项目投资30亿元以上。

统筹推进新区拓展和旧城改造，苍海新区基本实现环湖交通畅通目标，红岭新区建设、南岸片区开发稳步推进，长洲岛综合整治开发工程正式启动，百花冲片区棚改工作创造了新经验。加快县域城镇化发展，苍梧新县城规划、征地和重点项目建设稳步推进，岑溪、藤县和蒙山新型城镇化建设取得新进展。生态环保方面。

加强大气污染防治、直排口整治和污水垃圾处理设施建设，全市空气质量优良率达94.5%，集中式饮用水水源水质达标率100%，城镇污水处理率和生活垃圾无害化处理率分别达86%和100%。

深入推进美丽梧州·生态乡村建设，全面完成村屯绿化、饮水净化、道路硬化任务。

大力实施“绿满八桂”造林绿化工程，完成植树造林面积近22万亩，全市森林覆盖率达75.85%，森林立木蓄积量5100多万立方米，均在全区保持领先地位。蒙山县被纳入国家重点生态功能区，并通过了国家级美丽乡村建设标准化试点验收。

以推进供给侧结构性改革为主线激发新活力。深入落实“三去一降一补”重点任务，加强重点领域改革攻坚，激发市场活力和社会创新力。综合施策降低企业成本。贯彻落实自治区降成本“41条”，从电价、融资、税费、人工、物流和降低制度性交易成本等方面着手，累计为企业减负7.6亿元以上。优化传统产业供给结构。深入实施质量品牌提升行动，3个产品荣获“广西名牌产品”荣誉称号，3个品牌获评为“广西特产行销全国核心品牌”，梧州神冠蛋白肠衣有限公司荣获第三届自治区主席质量奖，藤县获得“中国钛白之乡”称号。淘汰了一批实心砖生产企业，全年压减炼钢产能185万吨。

加快房地产业去库存。结合实际出台了房地产去库存6项具体措施，城区商品房去库存周期从原来预期的36个月下降为17个月左右。强化金融风险防控。建立防范化解涉企金融风险工作机制，成立专门处置小组，避免相关金融机构和企业损失100多亿元。深化“放管服”改革。取消行政审批事项40项，集中清理前置中介服务事项95项，对外公布了第二批40个部门的权力清单、责任清单。在全区首批组建了城市管理行政执法局，市行政审批局筹备工作全部就绪。农村金融改革成效显著，信用户、信用村、信用镇创建工作走在全区前列。商事制度、国资国企、工业用地市场化配置等重点改革事项稳步推进。

深入推进向东开放合作。加强城市战略合作。与茂名市签署战略合作协议，与广州、肇庆、云浮、茂名分别召开市长联席会议，成功举办了西江经济带城市共同体及市长联席会议第一次会议，把珠西沿线城市合作推向新的高度。组团到香港、澳门考察学习，达成众多合作事项。加快粤桂合作特别试验区建设。争取自治区出台了《粤桂合作特别试验区改革创新总体方案》和3个配套文件。加快推进路网、管网等基础设施建设，全年完成土地平整1700多亩，开工建设厂房面积超过10万平方米。创新投资融资机制，与金融机构合作设立产业基金、创业基金130多亿元。试验区新增注册企业51家，企业总数达到230多家。加大招商引资力度。

组织举办了30多场招商活动，引进世界500强和中国500强企业投资项目3个，全市完成招商引资区外到位资金突破600亿元。

成功举办了第13届梧州宝石节、2016桂台（梧州）茶产业创新发展研讨会暨茶叶交易会等节庆活动，举办六堡茶产业发展高峰论坛、医药产业政策分享会等宣传推介会。

集中力量打好脱贫攻坚第一战。

建立四级联动帮扶工作机制，结对帮扶贫困户58000多户，实现贫困户结对全覆盖。认真落实中央和自治区各项扶贫政策，出台了20个扶贫配套政策性文件。全面完成精准识别建档立卡工作。强化扶贫资金支持保障，各级财政共筹措专项扶贫资金6亿元，累计发放扶贫小额贷款金额11.2亿元。加大产业扶贫开发力度，投入资金4000多万元，扶持发展了一批特色种养业项目。

扎实推进易地扶贫搬迁8个集中安置点建设，项目投资完成年度计划的70%以上，入住率达到60%以上，工作进度排全区第1位。加快贫困村路、水、电等基础设施建设，其中投入资金近2.5亿元，完成道路硬化近800公里。

加快推进以改善民生为重点的社会建设。全面完成了11大类民生实事工程项目，实际投资超过45亿元。深入实施教育发展十大重点工程，学前教育三年毛入园率、九年义务教育巩固率、高中阶段教育毛入学率分别达82.2%、97.4%和88.9%，万秀区、岑溪市义务教育均衡发展分别通过国家和自治区评估验收。深化医药卫生体制改革，公立医院综合改革工作顺利推进，新农合实现市级统筹并完成与居民医保制度整合。全面两孩政策稳步实施。认真落实促进就业创业政策措施，全市城乡新增就业7.7万人次，城镇登记失业率为2.95%。落实企业职工基本养老保险资金缺口责任分担机制，企业退休人员基本养老金月人均增加155元。完善养老服务体系，市云家庭服务中心建成投用，养老服务业综合改革试验区建设和服务机构公建民营试点工作顺利推进。深入实施文化惠民工程，建设村级公共服务中心50个，更新农家书屋300多家，梧州粤剧文化的品牌影响力不断扩大。

广泛开展全民健身运动，成功举办全国男子水球冠军赛、第十二届市运会暨第二届全民健身运动等赛事活动，藤县世界狮王争霸赛被命名为广西少数民族传统体育品牌赛事项目。加强和创新社会治理，深入开展“平安梧州”建设，被誉为“梧州模式”的一村（社区）一法律顾问工作全面推广，社会治安防控、应急管理、食品药品监管、安全生产、气象、防灾减灾等工作不断加强，社会大局保持和谐稳定。妥善应对苍梧县“7·31”地震灾害，最大限度减少了灾害损失，人民群众无一伤亡。

持续加强政府自身建设。

深入开展“两学一做”学习教育，在政府系统组织开展“多下基层、多解难题、敢于担当”活动，市层面共解决制约发展和群众反映集中的难点热点问题300多个，推动作风转变和履职能力提升。以“两个责任”清单全覆盖为抓手，推动政府系统落实全面从严治党责任。建立健全法治政府建设制度体系，制定了依法行政考核指标和考评办法，对50个部门和单位进行了考评，促进依法行政水平提升。积极推进政府立法工作，制定印发了《梧州市政府立法工作操作规范》，组织起草了《梧州市城市市容和环境卫生管理条例（草案）》。进一步推进简政放权，部门权力事项、行政审批事项精简率分别达47%、51%，非行政许可事项实现“清零”。坚决贯彻落实中央八项规定精神和国务院“约法三章”，加强重点领域行政监察和审计监督，全面公开财政预决算、部门预决算和“三公”经费预决算，积极推行政府购买服务，政府采购节约率达8.6%。认真执行市人大及其常委会的决议决定，自觉接受人大法律监督、政协民主监督和社会公众监督，高度重视和高质量办好了人大代表建议和政协委员提案。特别是市十四届政府班子履职以来，切实在转作风、重规范、提效能上下功夫，修改完善了《梧州市人民政府工作规则》，完善行政决策机制，强化权力运行机制和监督机制，着力提高依法行政、全面履职的能力和水平，树立起风清气正劲足的政府机关形象。

一年来，国防动员和后备力量建设不断加强，军民融合、人民防空、双拥共建等工作取得新成果。编制、审计、民族宗教、外事侨务、港澳台、统计、物价、农机、移民等工作取得新进步，工会、共青团、妇女儿童、残联、科协、社会科学、慈善、档案、地方志等工作取得新成效，中直、区直驻梧各单位为梧州经济社会发展做出新贡献。

各位代表，过去一年取得的各方面成绩，是自治区党委、政府和市委正确领导的结果，得益于市人大、市政协的监督和支持，凝聚了全市人民的辛勤与智慧。在此，我代表市人民政府，向全市各条战线的干部群众，向人大代表、政协委员，向各民主党派、工商联、无党派人士、人民团体，向离退休老领导、老同志，向驻梧部队、武警官兵、公安干警，向所有关心支持梧州发展的社会各界人士，表示崇高的敬意和衷心的感谢！

在总结成绩的同时，我们也清醒地看到发展中的困难和问题：经济下行压力仍然较大，结构调整任务繁重，新的增长动能尚未整体形成；实体经济盈利能力不强，财政收入进入低增长期，增收困难加大；城乡发展不均衡，民生社会事业仍然存在不少短板；少数干部创新精神、担当意识有待增强，“庸懒散拖”等现象仍一定程度存在，等等。对此，我们将认真采取措施，切实加以解决。

2017年工作安排

今年是梧州建城2200周年，秉承厚重历史文化，续写现代文明新篇，推动千年古城崛起，这是历史赋予我们的责任。当前，我国已进入“十三五”规划实施和深化供给侧结构性改革的重要时期，经济发展长期向好的基本面没有变，积极的因素不断涌现；自治区深入推进“双核驱动”战略，加快构建两广一体化发展，新的发展动能不断释放。我市既处于重要战略机遇期，又处于爬坡过坎的关键期，必须保持开拓进取的锐气和昂扬向上的士气，战略上坚持持久战，战术上打好攻坚战，努力在新常态中抢抓新机遇，实现新发展。

今年政府工作的总体要求是：全面贯彻党的十八大和十八届三中、四中、五中、六中全会精神，深入贯彻习近平总书记系列重要讲话精神，坚持稳中求进工作总基调，牢固树立和贯彻落实发展新理念，围绕市第十三次党代会和全市经济工作会议的部署要求，坚持以做大增量和提升质量为中心，以推进供给侧结构性改革为主线，以改革创新和向东开放为动力，以“三年一工程”为抓手，切实做好稳增长、促改革、调结构、惠民生、防风险各项工作，努力保持略高于全区平均水平的发展速度，促进经济平稳健康发展和社会和谐稳定，加快推动“三个实现”奋斗目标落实，以优异成绩迎接党的十九大胜利召开。

初步考虑今年全市经济社会发展的主要预期目标是：地区生产总值增长8%左右，固定资产投资增长9%以上，规模以上工业增加值增长8.5%，财政收入增长3%，社会消费品零售总额增长10%，进出口总额增长5%，居民人均可支配收入增长8%，城镇登记失业率控制在4.5%以内，节能降碳减排等约束性指标达到自治区的要求。

为完成上述目标任务，重点抓好以下八方面的工作：

一、发展实体经济，夯实稳定增长根基。实体经济是发展的基础，也是实现转型升级的主要载体。要把做好实体经济作为适应引领新常态的关键举措，营造全社会关心支持实体经济发展的良好氛围，不断做大增量、优化存量、提升质量。

推进工业转型升级。坚持项目集中园区、产业集中发展，通过纵向延伸拉长制造链条，横向拓展完善配套功能，集中力量打造高度关联、相互支撑的产业链，实现集群效应和规模效益。再生园区重点推进再生铜项目复产等12个续建项目，新开工年产10万吨再生锌等10个项目，竣工投产骆驼蓄电池等8个项目；不锈钢园区重点推进不锈钢精深加工园中园项目，新开工不锈钢板材项目；梧州高新区重点推进光纤陶瓷插芯精密制造等16个续建项目，新开工半导体LED芯片封装等14个项目。支持各县（市、区）加快发展特色产业园区。同时，加强重点产业园区基础设施和公共服务设施建设，全面推进临港经济区等新开发园区建设，提升园区的吸引力和承载力。持续开展质量品牌提升行动，鼓励再生资源、食品、医药、轻工、陶瓷建材等特色产业参与品牌创建，支持老牌企业技术创新和品牌建设，提高梧州制造的知名度和美誉度。

培育发展新兴产业。

以节能环保、生物制药、电子信息、新材料、新能源等新兴产业为突破口，抓好项目引进和产业配套，建设一批服务功能突出、产业特色鲜明的新兴产业基地。

以中盟医药产业园为载体，加快发展生物制药产业。推进智慧家电生产基地、中兴云数据中心、溯源大数据应用等电子信息产业项目建设。

大力发展特色农业。

着力推进水稻生产全程机械化，确保粮食总产量稳定在81万吨以上。深化农业供给侧结构性改革，发展壮大水果、中草药、六堡茶等特色种植业，以及生猪、三黄鸡、网箱鱼、蜜蜂等优势养殖业，着力增加有机绿色、安全优质农产品供给。继续抓好现代特色农业示范区建设，大力培育新型农业经营主体，各县（市、区）成立2家以上农民以土地入股经营的农业合作社。发展农产品深加工，新增农业产业化龙头企业3家以上。加快培育建设农产品专业市场，推进“农超对接”“批零对接”“农消对接”等，拓宽农产品流通渠道。加强农业区域合作，与云浮市共同推动粤桂合作现代生态循环农业示范区项目启动，与茂名市共同推动梧茂农产品物流集散地开工建设。

大力发展林下经济，加大政策扶持力度，培育“龙头企业(专业合作社）＋基地＋农户”的发展模式，带动农民参与林下经济发展，实现林下经济产值75亿元以上。

提速发展服务业。推进市商贸物流园区等13个服务业重点项目建设，培育发展冷链物流、仓储配送和各类专业市场，优化重要交通节点间的运输方式，加快构建高效快捷的现代物流体系。推进苍海新区中央商务区、红岭中央商务区等城市综合体建设，促进传统商业转型发展。深入实施电子商务“十百千万”工程，推进同阿里巴巴集团的合作，加快推动农村淘宝等项目落地，共同打造县域电商，推动电子商务进农村、进社区。

以岭南生态养生新城建设为重点，实施一批养生养老、疗养康乐项目，加快发展大健康产业。结合创建历史文化名城，加快推进“梧州岭南文化休闲旅游名城”建设，进一步完善旅游公共服务基础设施，打造梧州骑楼城等龙头景区，加快“三总府”恢复重建，支持石表山创建5A级景区及李济深故居、天龙顶山地公园等创建4A级景区，支持泗州岛、大自然现代农业园等创建广西四星级乡村旅游区，支持蒙山打造特色旅游名县，推进蒙山镇、六堡镇、南渡镇等一批特色小镇建设。

增强科技创新能力。梧州高新区要全力创建国家级高新区。积极实施发明专利“双倍增计划”，深化与知名高校、科研机构的合作，重点推进中德高等职业技术学院、科技创业服务中心、医药科技企业孵化器、农业科技孵化园、梧州学院大学生综合发展中心等创新平台建设，支持中恒、神冠创建国家工程技术研究中心。大力支持六堡茶、生物医药、船用机械等优势产业的创新研究，着力打造六堡茶产品国家级质检中心，建设新能源电动汽车产品、陶瓷产品、不锈钢产品等一批自治区级质检中心。加快科研成果转化和高新技术企业发展，新增国家级高新技术企业10家。强化科技创新政策扶持，优化财政科技经费投入和管理，培育引进高端创新人才。

加大服务企业力度。继续贯彻落实自治区41条降成本政策措施，清理一批附加条款和前置条件。研究出台企业分类评价制度，在电价、资金、市场营销、品牌评比等方面对企业实施分类扶持，鼓励支持企业上规。鼓励公共资源交易、公共设施投资等领域优先使用本地产品，以本地市场资源扶助企业发展。加大金融支持实体经济力度，力争金融机构在我市投放的新增贷款达到80亿元，全社会融资总量突破1200亿元。加大“惠企贷”支持力度，建立和完善全市统一的政府性融资担保体系，实现融资担保业务在市、县两级的全覆盖，切实解决小微企业融资难、融资贵问题。管理好产业投资引导基金，鼓励股权融资、债券融资，争取10家以上企业在区域性股权交易市场挂牌。加强社会诚信体系建设，及时化解企业“两链”风险，切实维护良好的金融生态环境。

二、扩大有效需求，强化稳定增长支撑。坚持市场导向，以新供给带动和释放新需求，以扩大有效需求倒逼供给升级，实现稳增长和调结构互为支撑、互促共进。

狠抓有效投资。

加大项目攻坚力度，力争年内完成固定资产投资1280亿元以上，其中重大项目投资400亿元以上。交通方面，建成梧州至柳州高速公路，大力推进西江机场、贺州至巴马高速公路蒙山段、荔浦至玉林高速公路蒙山段、环城高速公路、207国道东绕城过境一级公路、藤县津北至梧州三龙公路、龙圩综合客运枢纽等续建项目建设，推动岑溪至罗定铁路实质性复工，力争开工建设梧州至信都高速公路，推进洛湛铁路永州经梧州至玉林段电气化改造、塘源综合码头等项目前期工作。

水利方面，深入开展防洪工程建设会战，加快推进苍海新区水利工程、长洲南北堤以及高旺、塘源、平浪、三龙等防洪堤项目建设。

能源方面，加快推进220千伏禤州变电工程等5个电网基建项目建设，加快推动220千伏思扶变电工程等项目前期工作。在扩大基础设施投资的同时，要采取措施促进工业技改投资和激活民间投资。深化技改投资审批制度改革，对纳入自治区技改目录清单的项目，备案办理“零前置”，一般行业节能评估实行“零审批”，促进企业投资意愿回升，确保技改投资增长10%以上。滚动推出一批PPP项目，开工建设市静脉产业园、粤桂试验区社学片区地下综合管廊等项目。

培育消费热点。

加大梧州名特优商标品牌建设、保护和推广力度，积极培育国家地理标志商标，开展“梧州特产行销全国”促销活动，组织企业参加各类大型会展活动，以更加优质的产品、更加丰富的业态、更加便利的服务，引领和创造消费需求。完善促进消费政策体系，优化消费市场环境，培育发展文化、体育、健康、养老、教育培训等消费热点。引导城市综合体业态提升，推进乡镇商贸中心和社区商业中心建设。

加强区域旅游合作，加快打造精品旅游线路，培育发展户外运动、休闲养生、民俗民宿等新兴旅游业态，开展“岭南风情•梧州行”等旅游促销活动，力争旅游总收入突破200亿元。

稳定外贸增长。积极实施第二轮“加工贸易倍增计划”，加快培育一批重点加工贸易企业，推进新型外贸综合服务平台与本地企业合作，提升中小外贸企业的综合竞争力。加快跨境电商产业园和大龙网跨境电商平台建设，推进宝石城市场采购贸易方式试点工作，不断形成新的外贸增长点。促进CEPA落地，深化与港澳地区在服务领域的合作。继续实施集装箱奖励和定期航班政策，提高梧州口岸综合竞争力，降低企业物流成本。

三、深化区域合作，以向东开放带动全面开放。加快向先进生产力靠拢，不断提升开放合作水平，努力形成深度融合的开放合作格局。

全面务实推进区域合作。深化与广州、佛山、肇庆、云浮、茂名等城市的战略合作，重点推动柳肇铁路、岑罗铁路、温氏现代农业等41项重大合作事项。积极参与南广高铁和粤桂黔高铁经济带建设，健全西江经济带城市共同体及市长联席会议制度，强化与周边城市在基础设施、产业发展等方面的对接合作与共建共享。深化与香港、澳门的交流合作，抓好供港、供澳农产品基地建设，积极为港澳客商来梧投资提供便利化服务。积极参与“一带一路”建设，引导本地企业立足产品和资源优势拓展海外市场，鼓励外资企业在梧设立营销网络、生产基地或总部经济。

继续举办好梧州宝石节、六堡茶博览会等重大展会，提升梧州城市影响力。

大力推进粤桂合作特别试验区建设。全面落实《粤桂合作特别试验区改革创新总体方案》和3个配套文件，完成行政审批制度、金融体制、人事管理制度等专项改革任务。开工建设北斗导航、大华智慧信息、红牌智慧家电等18个项目，力争丰歌低碳科学建造基地、丰冠风光能发电系统应用等8个在建项目建成投产。继续推进基础设施建设，实施好一批路网、管网、电网项目。全面落实《两广金融改革创新综合试验区总体方案》和行动计划，发起2支以上产业基金或创业基金。加快梧州综合保税区申报工作。

努力提高招商引资水平。实行招商引资“一把手”工程，着力营造全市上下关心支持推动招商引资工作的浓厚氛围。组建外资服务中心和外商投诉中心，强化招商引资项目“一对一”代办服务，提高行政审批效率，加强正向激励和督查考核，狠抓签约项目落地；畅通客商反馈和投诉渠道，及时兑现招商引资承诺，着力解决好客商反映强烈的突出问题。加强精准招商，突出产业链招商，统筹推进驻点招商、专题招商、以商招商、专业小分队招商，提高招商引资规模和质量。推进招商项目库、企业名录库、实施项目库和投资促进网建设。力争全年招商引资到位资金达到660亿元以上。

四、坚持改革引领，激发经济社会发展活力。强化问题导向，突出重点，扭住关键，精准发力，推动各项改革举措落地生根、见到实效。

深入推进供给侧结构性改革。去产能方面，重点推动再生资源、再生不锈钢、陶瓷建材等产业升级换代，切实防止新增过剩和落后产能；推进“僵尸企业”清理处置工作，确保有序引导、平稳退出。去库存方面，积极推进棚户区改造货币化安置，鼓励和支持农民工购房，积极探索公租房以购代建，逐步建立购租并举的住房制度。去杠杆方面，加强金融风险防控，鼓励企业直接融资；健全完善政府性债务管理系统，加大政府存量债务处置力度。降成本方面，深入推进“放管服”改革，打好减负“组合拳”，力争全市企业综合成本下降5%—8%。补短板方面，从经济社会发展的关键环节和民生领域着手，加强基础设施建设和公共服务供给，使就业、教育、医疗、文化、社保、住房等体系更加健全。

深化重点领域改革。深化“放管服”改革，加快推进相对集中行政许可权改革试点，行政审批局全面运行，实现“一颗印章管审批”；继续做好行政审批及中介服务事项的取消、下放，实现权责清单“两单融合”；继续深化商事制度改革，巩固和扩大“六证合一、一照一码”改革成果，全面推行“双随机、一公开”监管模式。推进国资国企改革，探索以管资本为主的国资监管新机制，促进国有资产保值增值，加快推进政企分开、政资分开；积极稳妥发展混合所有制经济，加快建立现代企业制度。深化农村综合改革，全面铺开国有林场改革，加快农村土地承包经营权确权登记颁证工作，稳步推进农村土地“三权分置”，完成长洲区农村集体资产股份权能改革国家试点任务。深入推进“一套系统管信用”改革，积极创建国家社会信用体系建设示范城市。统筹推进财税、供销社、社保、医疗卫生等领域的改革。

深化要素市场改革。深化工业用地市场化配置改革国家试点工作，支持粤桂试验区实施“先租后让”“弹性出让”等更加灵活的供地方式。加强电力需求侧管理，推动售电市场化改革，扩大电力直接交易适用范围，让市场主体及城乡居民获得更多改革红利。健全社会资本参与公共项目投资运营的模式和流程，建立合理回报机制，进一步加大PPP模式推广应用力度。

五、加大统筹力度，促进城乡协调发展。以规划为引领，以项目为支撑，全面推进以人为核心的新型城镇化建设，努力提升城乡品质和一体化发展水平。

继续实施城市建设提升工程。以梧州建城2200周年为契机，筹集资金54亿元以上，组织实施一批城建项目建设，重点抓好基础设施、新区建设、旧城改造、安居工程、公共服务、环境优化等6大类项目，实现城市建设三年提升工程圆满收官。基础设施方面，西江三桥建成通车，推进西江四桥、城市骨干路网、公共停车场、海绵城市试点等项目建设，谋划储备一批地下综合管廊项目，实施一批城市配电网项目。新区建设方面，苍海新区重点推进东环大道、湿地公园等“路、景、城”项目建设；红岭新区重点推进路网、绿道及中央商务区等项目建设，全面提升玫瑰湖景观环境；积极推进南岸片区开发。旧城改造方面，重点推进工厂一路、新兴一路、富民、百花冲等棚户区改造，实施骑楼城保护性开发和长洲岛综合整治工程。环境优化方面，突出抓好城市环境综合治理，大力创建文明城市，加强交通秩序、街道经营秩序整治工作，打造一批管理精细、秩序井然、环境整洁的样板区域和示范道路。谋划举办形式多样的城市宣传推介活动，弘扬梧州深厚历史文化，提升作为梧州人的自豪感！

加快打造特色城镇群。强化中心城区与城镇协同发展，加快培育一批富有特色和活力的现代化城镇。支持苍梧县加快新县城建设，支持岑溪打造桂东南副中心城市，支持藤县撤县设市和建设产城融合、生态宜居的新兴城市，支持蒙山县打造广西特色旅游名县。积极培育产业强镇、历史文化名镇、特色小镇，完善城镇功能，增强人口和经济综合承载能力。

推进宜居乡村建设。完成农村垃圾治理两年攻坚任务，加快垃圾处理设施建设，改进垃圾分类收运体系，逐步实现生活垃圾减量化目标。在巩固提升“清洁乡村” “生态乡村”的基础上，大力推进“宜居乡村”建设活动，重点实施产业富民、服务惠民、基础便民等专项活动。充分整合各类资金，推进农村基础设施建设，建成一批示范村，切实改善农村生产生活条件。各级共同努力，大力实施“第一公里·桥”攻坚战，新建或改造加固一批农村桥梁，逐步淘汰“横水渡”、简易桥和“铁索桥”，逐年解决农村群众出门“第一公里”的通畅和安全问题。

六、坚持绿色发展理念，保持生态环境优势。协调处理好环境保护与经济发展的关系，既要金山银山，更要绿水青山。

加大环境整治力度。严格执行“大气十条”，继续加强工地施工、道路扬尘等污染治理及重点大气污染源的监管力度，加快淘汰黄标车和老旧车，确保氮氧化物、PM2.5、PM10等指标控制在自治区下达范围内。严格执行“水十条”，全面推行“河长制”，加大饮用水水源地保护，启动市区饮用水水源取水口迁移工作，彻底整治市区污水直排口，清理水源地保护区畜禽养殖，确保水质达标率95%以上。加快城镇污水处理设施建设，完成4个镇级污水处理厂及配套管网建设任务。严格执行“土十条”，加强农业面源污染防治。加快推进自然保护区和功能区划定，严格限制生态敏感区的开发建设活动。

推动绿色低碳发展。加快 “国家城市矿产”示范基地建设，推进园区循环化改造，带动循环再生产业发展。提高建筑节能标准，推广绿色建筑和建材。严把环保准入关口，逐步淘汰和控制新建高耗能项目。抓好工业、交通等领域低碳节能技术应用，推广新能源汽车应用公共交通领域，鼓励低碳消费、绿色出行。

巩固扩大生态优势。坚持生态效果与景观效果相统一，实施森林质量提升、城市绿化增量提质、森林公园和湿地公园扩面工程，巩固和扩大“国家森林城市”“国家园林城市”创建成果。大力开展造林绿化活动，推进生态公益林、经济林产业建设，发展林下经济、森林产业。加强江河生态屏障、生态廊道体系建设，大力推进水生态文明建设工作。

七、致力共建共享，切实保障和改善民生。践行以人民为中心的发展思想，坚持普惠性、均等化、可持续发展，不断补强民生短板，让发展成果更多更公平地惠及全市人民。

持续打好脱贫攻坚战。深入实施精准脱贫“七个一批”“十大行动”，在巩固2016年脱贫成果的同时，确保今年实现7.5万贫困人口脱贫、68个贫困村和蒙山县脱贫摘帽。推进贫困村屯级道路、水利、网络、农村电网、贫困户危房改造等项目建设。全力抓好易地扶贫搬迁工作，强化规划引导和用地资金保障，全年完成搬迁安置贫困人口9900人以上。支持贫困村因地制宜发展特色优势产业，加快形成“一村一业、一村一品”格局。继续做好扶贫小额信贷工作，引导贫困户以土地、财政扶持资金入股市场经济组织运营，探索资产收益脱贫新模式。

落实贫困户子女各阶段学生资助政策，加强农村贫困劳动力创业就业帮扶。深入开展“一帮一联”活动，增强贫困群众主体意识和脱贫信心，提升贫困群众自身“造血”能力。

强化基本民生保障。筹措资金45亿元以上，继续集中力量办好一批民生实事。落实自治区就业创业工作8条措施，加快农民创业平台建设，抓好高校毕业生创业就业。全年城乡新增就业7万人次，技能培训2万人。推进社保扩面和“五险合一”征缴，抓好机关事业单位养老保险制度改革，进一步做好企业职工基本养老保险工作。落实城乡居民基本医疗保险制度，实现社保“一卡通”。推行企业工资集体协商制度，落实最低工资保障和增长机制。加强城区劳动仲裁实体化建设，打击拖欠农民工工资行为。深入开展农民增收行动。健全完善以城乡居民最低生活保障和特困人员供养为主体，以医疗救助、教育救助、住房救助、临时救助等专项制度为补充的社会救助体系，保障困难群众基本生活。加快推进养老服务业综合改革试验区建设，构建多层次养老服务体系。坚持和完善计划生育目标管理责任制，强化基层基础，加强生育保障，促进家庭发展。

优化公共服务供给。加大教育基础设施投入，筹措资金2.9亿元新建和改扩建一批中小学校。加快推进第三期学前教育三年行动计划和义务教育均衡发展、高中阶段教育突破发展工程，继续抓好“控辍保学”工作。继续支持梧州学院更名为梧州大学相关工作，积极筹办梧州医学高等专科学校。全面开展城市公立医院综合改革试点，扎实推进县级公立医院综合改革工作。优化城市医疗资源布局，推动市人民医院新院区投入使用并实现合作发展，推进市工人医院和市妇幼保健院扩建等项目建设。积极推进基层医疗卫生机构能力建设，深入实施基层中医药服务能力提升工程。

完善公共文化服务体系，加快县（市、区）图书馆、文化馆和乡镇文化站标准化建设。

推进苍海文化中心、梧州粤剧保护与传承基地等项目建设，办好第三届广西（梧州）粤剧节。实施竞技体育重振工程和业余体校强基工程，提高竞技体育水平。完善社区体育健身设施，改进公共体育设施管理办法，打造城镇居民“15分钟健身圈”。

办好“一带一路”国际青少年足球邀请赛、全国青少年足球锦标赛等大型体育赛事。

提升社会治理实效。深入推进“平安梧州”建设，构建立体化社会治安防控体系。深化公安改革和社会组织管理制度改革，加强社区（村）管理服务能力建设。扎实开展“七五”普法，推进各级公共法律服务中心（站）规范化建设。畅通群众诉求表达渠道，切实维护群众的合法权益。完善应急体系建设，加强防汛抗旱和地质灾害防治工作，提高防灾减灾救灾能力。统筹推进安全生产领域改革，强化政府领导责任、部门监管责任和企业主体责任落实，深化重点行业领域安全生产专项整治，努力实现各类事故稳中有降，杜绝重特大事故发生。大力夯实“四有两责”监管基础，严防严管严控食品安全风险，创建广西食品安全城市。推进国防教育、国防动员和后备力量建设，推动军民融合深度发展。深入开展双拥共建活动，争创全国双拥模范城。继续做好民族宗教、外事侨务、对台、统计、审计、人防、气象、地震、档案、地方志、扫黄打非等工作，支持工会、共青团、妇联等群团组织发挥更大作用。

八、加强自身建设，打造人民满意政府。

切实增强政治意识、大局意识、核心意识、看齐意识，按照营造风清气正的政治生态要求，以全面从严治党统领政府各项工作，不断提高政府施政能力和服务水平，加快建设法治型、服务型、效能型和廉洁型政府，推进政府治理能力现代化。

必须依法行政。自觉接受人大法律监督、工作监督和政协民主监督，认真办理人大代表建议和政协委员提案，认真听取民主党派、工商联、无党派人士和各人民团体的意见。健全依法科学民主决策机制，严格遵守重大行政决策法定程序。加强政府立法工作，健全完善政府立法机制，深入推进城市建设与管理、环境保护等领域的立法工作。完善行政执法程序，规范执法行为，推动综合行政执法向乡镇延伸，实现“四所合一”“多所合一”。全面推进政务公开，自觉接受群众监督，主动回应社会关切，充分体现人民意愿。

必须务实担当。始终保持奋发有为的精神状态，把推动梧州发展的责任扛在肩上，主动作为，务实担当，在“真”字上花力气，在“实”字上下工夫。不断完善抓落实的工作机制，把抓落实作为衡量工作成效的重要标准，营造崇尚实干的氛围，让实干在梧州蔚然成风，推动梧州各项工作干在实处、走在前列。

必须开拓创新。跳出思维定势，克服工作惯性，摆脱路径依赖，以改革创新破解发展难题。始终保持开拓创新的锐气，把梧州置于全新发展坐标下，改革参与上先行一步，工作落实上主动一步，创造更好的条件、整合更大的资源、争取更多的项目，赢得梧州发展新机遇、新空间、新前景。

必须廉洁从政。坚持把纪律挺在前面，全面落实党风廉政建设责任制，严格执行党的廉洁自律准则、纪律处分条例、问责条例和监督条例。进一步完善政府投资项目、土地出让、产权交易、政府采购等制度，进一步规范公共资金、公共资源和国有资产监管。切实加强行政监察和审计监督，确保权力在阳光下廉洁高效运行。

各位代表！340万梧州人民对美好生活的向往，就是我们矢志不渝的奋斗目标。让我们紧密团结在以习近平同志为核心的党中央周围，在自治区党委、政府和市委的坚强领导下，紧紧依靠全市人民，以更加饱满的激情、更加昂扬的斗志、更加务实的作风，锐意进取、奋力拼搏，以优异的成绩迎接党的十九大胜利召开！
